# The genetic effects of the dopamine D1 receptor gene on chicken egg production and broodiness traits

**DOI:** 10.1186/1471-2156-11-17

**Published:** 2010-03-03

**Authors:** Haiping Xu, Xu Shen, Min Zhou, Meixia Fang, Hua Zeng, Qinghua Nie, Xiquan Zhang

**Affiliations:** 1 Department of Animal Genetics, Breeding and Reproduction, College of Animal Science, South China Agricultural University, Guangzhou 510642, Guangdong, China; 2 Biotechnology Institute, Jiang Xi Education College, Nanchang 330029, Jiangxi, China; 3 Department of Laboratory Animal Science, Medical College of Jinan University, Guangzhou 510632, Guangdong, China

## Abstract

**Background:**

The elevation of egg production and the inhibition of incubation behavior are the aims of modern poultry production. Prolactin (*PRL*) gene is confirmed to be critical for the onset and maintenance of these reproductive behaviors in birds. Through PRL, dopamine D1 receptor (DRD1) was also involved in the regulation of chicken reproductive behavior. However, the genetic effects of this gene on chicken egg production and broodiness have not been studied extensively. The objective of this research was to evaluate the genetic effects of the *DRD1 *gene on chicken egg production and broodiness traits.

**Results:**

In this study, the chicken *DRD1 *gene was screened for the polymorphisms by cloning and sequencing and 29 variations were identified in 3,342 bp length of this gene. Seven single nucleotide polymorphism (SNPs) among these variations, including a non-synonymous mutation (A+505G, Ser169Gly), were located in the coding region and were chosen to analyze their association with chicken egg production and broodiness traits in 644 Ningdu Sanhuang individuals. Two SNPs, G+123A and C+1107T, were significantly associated with chicken broody frequency (P < 0.05). Significant association was also found between the G+1065A - C+1107T haplotypes and chicken broody frequency (P < 0.05). In addition, the haplotypes of G+123A and T+198C were significantly associated with weight of first egg (EW) (P = 0.03). On the other hand, the distribution of the *DRD1 *mRNA was observed and the expression difference was compared between broodiness and non-broodiness chickens. The *DRD1 *mRNA was predominantly expressed in subcutaneous fat and abdominal fat of non-broodiness chicken, and then in heart, kidney, oviduct, glandular stomach, hypothalamus, and pituitary. In subcutaneous fat and abdominal fat, the level of non-broodiness was 26 to 28 times higher than that of broodiness. In pituitary, it was 5-fold higher. In heart, oviduct, and kidney, a 2-3 times decrease from non-broodiness to broodiness was displayed. In glandular stomach and hypothalamus, the level seen in non-broodiness and broodiness was almost the same.

**Conclusion:**

The polymorphisms of the *DRD1 *gene and their haplotypes were associated with chicken broody frequency and some egg production traits. The mRNA distribution was significant different between broodiness and non-broodiness chickens.

## Background

In modern poultry production, chickens are constantly selected for a high rate and persistency of egg laying. However, incubation behavior usually results in the regression of ovary and the cessation of egg laying [1,2]. In addition, following the adoption of artificial incubation technology, incubation behavior is no longer required in chicken production. Therefore the inhibition or eradication of incubation behavior has received much attention as a potential target of egg production improvement. Recently with the development of molecular biological approaches, the genetic basis of broodiness has been extensively studied. It is a polygenic trait controlled by at least two dominant autosomal genes [3,4]. Although a study [2] to search the QTL of broodiness was carried out, to date no QTL was identified by genome-wide scan approach. On the other hand, many investigations about the genetic basis of egg production and broodiness have been performed through candidate gene analysis, especially for the prolactin (*PRL*) gene [5-8]. PRL is postulated to play a critical role in the onset and maintenance of incubation behavior in birds [9-12]. Some other factors, such as dopamine, have pivotal effects on PRL secretion [13-15].

Dopamine, an abundant neurotransmitter in the central nervous system and periphery, has been shown to play important roles in cognition, emotion, endocrine function, and hyperprolactinemia in mammals [16-19]. Its physiological effects are exerted through activating dopamine receptors. So far, at least five distinct dopamine receptors subtypes, DRD1-DRD5, have been identified and classically divided into two classes referred to as D1-like (DRD1 and DRD5) and D2-like (DRD2, DRD3, and DRD4) receptors based on their pharmacological, biochemical, and physiological differences [20-22]. All of these receptors are G protein-coupled receptors with 7 transmembrane domains. In avian, dopamine was demonstrated to be involved in both stimulating and inhibiting PRL secretion in the brain [23]. Dopamine stimulates PRL secretion via activating DRD1 at the hypothalamus level by operating through vasoactive intestinal peptide (VIP) [24-26]. And the inhibition effect of dopamine on PRL secretion is mediated through DRD2 receptors at the pituitary level [27,28]. Hens treated with dopamine receptor antagonist or receptor blocking agent resulted in terminated maintenance of broodiness by inhibiting secretion of PRL [29-31]. Sartsoongnoen also found that an association existed between DA neurons and the regulation of the reproductive system in Thai chickens [32]. All these studies suggested that dopamine receptor was involved in the regulation of avian reproductive behavior. In this study, the *DRD1 *gene was chosen as a candidate gene to analyze the genetic effect on chicken egg production and broodiness traits.

Like other D1-like members, the chicken *DRD1 *is an intronless gene and belongs to the rhodopsin family. It is located on chromosome 13 and contains an open reading frame of 1356 nucleotides encoding a protein of 451 amino acids [33]. Demchyshyn found that chicken *DRD1 *mRNA was predominantly expressed in the brain and to a much less extent in the kidney, whereas in other peripheral tissues such as, spleen, liver, heart and lung, no expression was detected by Northern blot analysis [34]. Recently, some studies demonstrated that the *DRD1 *gene was widely expressed in the hypothalamus and pituitary and the expression was correlated with the reproductive system in turkeys [35,36]. Schnell indicated that no significant difference was observed in hypothalamic expression of the *DRD1 *gene throughout the reproductive cycle, as well as in pituitary [35]. However, subsequent investigation showed that the hypothalamic expression increased in hyperprolactinemic incubating turkey hens [36].

Although the mRNA expression patterns of the *DRD1 *gene were partially reported in avian, the associations of these patterns and the *DRD1 *gene polymorphisms with chicken reproductive traits remained unclear. The aim of the present research was to screen polymorphisms in the *DRD1 *gene coding region and evaluate their genetic effects on chicken egg production and broodiness traits. Furthermore, the mRNA expression difference of this gene was investigated between broody and non-broody chickens.

## Methods

### Chicken Populations

A total of 24 unrelated chickens were used to identify the mutations in the *DRD1 *gene. They were from 6 populations (4 from each) including Red Jungle Fowls (RJF), Taihe Silkies (TS), Xinghua chickens (XH), Gushi chickens (GS), White Recessive Rock Broilers (WRR), and Leghorn Layers (LH). The detail information of the populations was shown in Table 1.

**Table 1:** The characterization of the populations used in this study

Populations	Origin	Production performance
Red Jungle Fowls (RJF)	Linshan County, Guangxi, China	Seasonal reproduction and broodiness; an egg-production of 60 per year.
Taihe Silkies (TS)	Taihe County, Jiangxi, China	A 70 to 80% incidence of broodiness; an egg-production of 70-80 per year.
Xinghua chickens (XH)	Fengkai County, Guangdong, China	A 70 to 80% incidence of broodiness; an egg-production of 60-90 per year.
Gushi chickens (GS)	Gushi County, Henan, China	A 10 to 20% incidence of broodiness; an egg-production of 141 per year.
White Recessive Rock Broilers (WRR)	Commercial broiler line imported from Kabir Co Ltd, Italy	No broodiness in cage; an egg-production of 180 per year.
Leghorn Layers (LH)	Commercial layer line derived from Italy	No broodiness; an egg-production of 250-300 per year.
Ningdu Sanhuang chickens (NDH)	Ningdu County, Jiangxi, China	A 50 to 60% incidence of broodiness; an egg-production of 110-130 per year.

The population for association study consisted of 644 female Ningdu Sanhuang (NDH) chickens obtained from Guangdong Wens Foodstuff Corporation Ltd. (Guangdong, China). These birds were randomly selected from 1477 unrelated birds. All NDH female chickens were fed with free access to water and feed to 77 d of age, and then changed to feed a corn-soy-bean-based diet with 15% CP and 2,900 kcal of ME/kg. All of them were exposed to a continuous 24 h photoperiod during the first 2 d of age, and then changed to and maintained under a daily light period of 16 h. They were reared in individual laying cages after 90 d of age. In this population, age of first egg (AFE), total egg number from 90 to 300 d of age (EN), total number of qualified eggs from 90 to 300 d of age (QEN), total number of oafish eggs from 90 to 300 d of age (OEN), and weight of first egg (EW) were observed. Qualified eggs were recognized with the criteria as: clean and smooth surface, ellipse shape with a big end and a small end, hard and complete eggshell, stable equilibrium color, similar in size and shape, a good sense of heaviness in hand, crisp noise and hardly breakup after mutual collision. Oafish eggs were abnormal eggs including double-yolk eggs, soft-shell eggs, ruptured eggs, rough-shell eggs, crack eggs, wrinkle eggs and so on.

In addition, from 90 d to 300 d of age the incubation behavior of chickens was observed and recorded at 16:00 pm everyday. The criteria for broody behaviors have been published elsewhere [8]. Briefly, when hens exhibited increased body temperature, nesting, incubating, feather loosening, lacking of luster throughout the body, specific clucking, being more defensive and aggressive, and lost their appetite, they were considered to be in broody. In the association analysis, two parameters, duration of broodiness (DB) and broody frequency (%), were investigated. DB was estimated by the total number of days a hen being in broody during the observation period. Broody frequency (%) was calculated by the percentage of broody chickens, and here individuals exhibiting obvious broody behavior for more than 1 d were identified as broody chickens considering enough sample numbers in statistics.

The distribution pattern of the *DRD1 *mRNA was studied in NDH female chickens. The expression differences were compared between 6 NDH chickens in broody and 6 individuals in non-broody in various kinds of tissues (heart, liver, spleen, lung, kidney, breast muscle, leg muscle, gizzard, glandular stomach, pituitary, hypothalamus, ovary, oviduct, duodenum, subcutaneous fat, and abdominal fat). All the tissues of broody chickens were taken at the midpoint (the fourth day after the onset of broody behavior) of broodiness and those of non-broody chickens were taken at the same day. All animal experiments were conducted in accordance with Law of the People's Republic of China on Animal Protection.

### DNA Extraction, PCR Amplification and PolymorphismIdentification

Genomic DNA was isolated from blood using the traditional method. Two pairs of primers (P1 and P2, shown in Table 2) used for the amplification of the chicken *DRD1 *gene were designed according to the published mRNA sequence [GenBank: NM_001144848] by Genetool software (http://www.biologysoft.com/; BioTools, Alberta, Canada). The polymorphisms of the whole *DRD1 *coding region were identified through the amplification of a 1970-bp fragment by primer P2 (Table 2). The PCR reactions were carried out in a total volume of 25 μL containing 50 ng of genomic DNA, 1 μM of each primer, 200 μM dNTP, 1.5 mM MgCl_2_, 1× PCR buffer and 1 U of Taq DNA polymerase (Sangon Biological Engineering Technology Company, Shanghai, China). The amplification was performed in a Eppendorf Mastercycler (Eppendorf Limited, Hamburg, Germany) under the following conditions: 94°C for 3 min; 35 cycles of 94°C for 30 s, n°C for 45 s and 72°C for 1 min; and 72°C for 10 min. PCR products were subjected to a 1% agarose gel electrophoresis and visualized in TFM-40 Ultraviolet Transilluminator (UVP Company, Cambridge, UK) by ethidium bromide staining. Subsequently, the DNA bands were excised from the gel and purified with OMEGA Gel Extraction Kit (OMEGA Bio-Tek Inc., GA, American), and then subcloned into the pMD18-T vector (TaKaRa Biotechnology Co., Ltd., Dalian, China). DNA sequencing was performed by the dideoxy chain-termination method using dye terminator cycle sequencing on Applied Biosystem model 3730 sequencer. The analysis of sequences was conducted by the software DNASTAR V 3.0 (http://www.biologysoft.com/; Steve ShearDown, 1998-2001 version reserved by DNASTAR Inc., Madison, Wisconsin, USA).

**Table 2:** Detail information for primers of the chicken *DRD1 *gene

Primer name	Primer sequence (5'→3')		Length^1 ^(bp)	Location^2^	AT^3 ^(°C)	Genotyping method
P1	F:CCGGTGAGTACCCTGCTTT R:GTGCTTTTCCTCTGCTTTGG		1504	-1961 ~ -458	59	/
P2	F:AGTGAAGAATTGCTCGCTGA R:GGTTTTGCTGGGTACACCTT		1970	-589 ~ +1381	57	/
P3	F:CACTATGGATGGGGAAGGGTTG R: GGCCACCCAGATGTTGCAAAATG		283	G+123A T+198C	62	*BseNI* *cfrI*
P4	F: CAGCCCATTCAGGTACGAGAGGA R: ATTCGACTCTTTGGGGCTGGAC		793	A+505G C+765T C+1011T G+1065A C+1107T	65.5	sequencing
P5	F: TTTCCTTCATCCCCGTGCAGCT R: GCTGCTTCTGTTGCCACTTGTGT		290	+458 ~ +747	63	/
P6 (actin)	F: CCCCAAAGCCAACAGAGAGA R: GGTGGTGAAGCTGTAGCCTCTC		274	/	63	/

^1^The length of PCR products; ^2^referred to the locations in the *DRD1 *gene, the first nucleotide of translation start codon was designated as +1. ^3^indicated annealing temperature.

### Genotyping of Polymorphisms

Primers (P3 and P4) used for genotyping of polymorphisms in the *DRD1 *coding region were described in Table 2. Genotypes of G+123A and T+198C were determined with PCR-RFLP method using genomic DNA from the 644 NDH individuals as templates. PCR products were subjected to digestion for 16 h at 37°C with the restriction enzyme BseNI and CfrI, respectively. The digestion mixture was composed of 8 μL PCR products, 1 × digestion buffer, and 3.0 U of each enzyme. Subsequently the fragments were visualized by TFM-40 Ultraviolet Transilluminator (UVP Company, Cambridge, UK) following separation in 2.5% agarose gels and staining with ethidium bromide. For the other SNPs in the coding region, genotyping was carried out by direct sequencing.

### RNA Extraction and cDNA Synthesis

Twelve NDH chickens, 6 broody individuals and 6 non-broody ones, were used in expression analysis. A total of 16 tissues, including heart, liver, spleen, lung, kidney, breast muscle, leg muscle, gizzard, glandular stomach, pituitary, hypothalamus, ovary, oviduct, duodenum, subcutaneous fat, and abdominal fat, were collected from each chicken. Among the 16 tissues, six tissues of pituitary, hypothalamus, ovary, oviduct, subcutaneous fat, and abdominal fat were important parts of chicken reproduction physiology system. Whereas other tissues including heart, liver, spleen, lung, kidney, breast muscle, leg muscle, gizzard, glandular stomach and duodenum were chosen to be the background tissues in chicken broodiness research. The dissected tissues were frozen in liquid nitrogen immediately and subsequently stored at -80°C until used. Total RNA was extracted with TRIzol reagent (Invitrogen, Carlsbad, CA, USA) following the manufacturer's instructions and then treated with DNase (Promega, Madison, WI, USA). The DNase reaction were composed of 1 μg of total RNA, 1 U RNase-free DNase, 1 μL 10 × Reaction Buffer and 7 μL nuclease-free water. The mixture was incubated at 37°C for 30 min followed by denaturation at 65°C for 10 min and snap cooled on ice for 2 min. The quality and purity of the RNA were checked by agarose gel electrophoresis and spectrophotometry. cDNA was synthesized in a final volume of 20 μL including 1 μg of total RNA, 1× MMLV Buffer, 1 mM of each dNTPs, 2.5 μM oligo (dT)_18_, 0.5 μL (40 U/μl) RNase inhibitor, 100 U MMLV SuperScript III reverse transcriptase (Invitrogen, Carlsbad, CA, USA). The reverse transcription was processed for 40 min at 42°C followed by heating for 5 min at 95°C and cooling on ice.

### Quantitative Real-Time PCR

Quantitative real-time PCR (qPCR) was performed with the ABI PRISM 7000 sequence detection system (Applied Biosystems, Foster City, CA, USA) using the SYBR Green PCR Master Mix. The obtained cDNAs were used as templates for qPCR amplification. Primers used for the qPCR were designed by Primer Express 2.0 software (Applied Biosystems, Foster City, CA, USA). A housekeeping gene, chicken *β-actin *gene [GenBank: LOC396526], was used as internal control. Therefore, two sets of primers (shown in Table 2), P5 and P6, were designed and used for the qPCR amplification of chicken *DRD1 *gene and chicken *β-actin *gene, respectively. Each reaction mixture contained 10 μL of SYBR Green PCR Master Mix, 2 μL of each primer (10 μM), 4 μL ultrapure RNase-free water and 2 μL of cDNA in a final volume of 20 μL. Standard amplification conditions were as follows: 95°C for 3 min; 40 cycles of 95°C for 30 s, 63°C for 30 s and 72°C for 40 s. Fluorescent signal were collected after the extension at 72°C in each cycle. Amplification of DRD1 and β-actin for each sample was run simultaneously in separate tubes and in duplicates. A negative control with sterile water as template was run for each primer in order to control the reagent contamination. The whole experiment was repeated at least twice. After amplification, dissociation curve analysis was conducted to ensure only one product. And then the product was sequenced to confirm amplification of the correct sequences.

### Statistical Analysis

#### Identification of the Chicken DRD1 Gene Polymorphisms and Prediction of the Transcription Factor Binding Sites in the 5' Flanking Region

DNAMAN (Lynnon Biosoft) was used for DNA contig assembly, sequence editing, and sequence translation. The identification of mutated sites was performed by MegAlign program of DNASTAR software (http://www.biologysoft.com/; Steve ShearDown, 1998-2001 version reserved by DNASTAR Inc., Madison, Wisconsin, USA). The potential transcription factor binding sites of the 5'-flanking region polymorphisms were predicted by two bioinformatic websites of http://motif.genome.jp and http://www.gene-regulation.com/pub/programs/alibaba2 following the setting parameters. The same results identified by the two websites were finally chosen.

#### Haplotype Inference and Marker -Trait Association Analysis

Hardy-Weinberg's equilibrium and the haplotype structure were analyzed by Haploview version 3.32 software http://www.broad.mit.edu/mpg/haploview/) [37]. Haplotypes were inferred based on the haplotype structure by the PHASE 2.0 software http://www.stat.washington.edu/stephens/software.html[38].

Association analysis of polymorphisms or haplotypes with egg production and broodiness traits were conducted by SAS GLM procedure (SAS Institute Inc., Cary, NC, USA) using the following model:

Where Y_ij _is an observation on the traits, μ is the overall population mean, G_i _is the effect of genotype, H_j _is the fixed effect of hatch and the e_ij _is the residual error. Multiple comparisons were performed with least squares means using the following procedure:

Where  is the least value, and . The results were presented as least square means ± standard error.

The comparisons of broody frequency among different genotypes or diplotypes in each site were evaluated by chi-square (χ^2^) tests performed on a 2 × 3 (or n) contingency table. A P ≤ 0.05 was considered statistically significant in all analysis.

#### Expression Analysis of DRD1 mRNA

Quantitative values were obtained from the threshold cycle (Ct) at which a significant increase in the magnitude of the signal generated by the PCR reaction started to be detected. The relative amount of chicken DRD1 mRNA in each tissue was calculated by the formula 2^-ΔΔCt ^[39], where ΔΔCt corresponded to the difference between the ΔCt measured for the mRNA level of each tissue and that measured for the mRNA level of reference tissue (the hypothalamus of non-broody chicken). Here ΔCt = Ct_target gene _- Ct_β-actin_. Results were expressed as means ± standard error means. Statistical analyses of differences in distinct tissues were processed with least square method by SAS 8.0 software (SAS Institute Inc., Cary, NC, USA). Significant differences between broody and non-broody chickens were detected using Student t test at a significance level of 0.05 in each tissue.

## Results

### Polymorphisms of the Chicken DRD1 Gene and Transcription Factor Binding Sites Prediction in the 5' Flanking Region

Twenty-seven single nucleotide polymorphisms (SNPs) and 2 indel variations (shown in Table 3) were identified in a total of 3,342 bp region of the chicken *DRD1 *gene, among which 7 SNPs were located in coding region and others in the 5' regulatory region. In the full region studied, on average every 115 bp generated one SNP. The polymorphism density of the coding region was just one SNP per 194 bp, and in the 5' regulatory region it was one per 89 bp. Among the 7 SNPs (shown in Table 4) in the coding region, only one SNP was non-synonymous mutation (A+505G, Ser169Gly) and located in the second extracellular domain. Three synonymous polymorphisms, C+1011T, G+1065A, and C+1107T, were located in the carboxylic tail of DRD1. The variations of G+123A, T+198C, and C+765T, occurred in transmembrane domain I, transmembrane domain II, and the third intracellular loop, respectively.

**Table 3:** Polymorphisms detected in the chicken *DRD1 *gene

No.	Variation^1^	region	No.	Variation^1^	region
1	A-1793C	5' regulatory region	16	A-647G	5' regulatory region
2	G-1735C	5' regulatory region	17	C-634G	5' regulatory region
3	C-1687T	5' regulatory region	18	A-570G	5' regulatory region
4	T-1679C	5' regulatory region	19	G-454A	5' regulatory region
5	G-1591C	5' regulatory region	20	T-349C	5' regulatory region
6	G-1463C	5' regulatory region	21	-225A indel	5' regulatory region
7	A-1412T	5' regulatory region	22	A-179T	5' regulatory region
8	-1157C indel	5' regulatory region	23	G+123A	Exon
9	C-1029T	5' regulatory region	24	T+198C	Exon
10	G-995A	5' regulatory region	25	A+505G	Exon
11	A-942G	5' regulatory region	26	C+765T	Exon
12	T-941C	5' regulatory region	27	C+1011T	Exon
13	A-910G	5' regulatory region	28	G+1065A	Exon
14	T-823G	5' regulatory region	29	C+1107T	Exon
15	C-684A	5' regulatory region			

^1^the first nucleotide of translation start codon was designated as +1.

**Table 4:** Detail information for polymorphisms in the *DRD1 *coding region

SNP^1^	AA Variation	Location^2^	SNP^1^	AA Variation	Location^2^
G+123A	Thr41Thr	TM I	C+1011T	Asn337Asn	CT
T+198C	Ala66Ala	TM II	G+1065A	Pro355Pro	CT
A+505G	Ser169Gly	Extracellular domain	C+1107T	Asn369Asn	CT
C+765T	Pro255Pro	The third intracellular loop			

^1^the first nucleotide of translation start codon was designated as +1. ^2^TM I = transmembrane domain I; TM II = transmembrane domain II; CT = the carboxylic tail.

The analysis of the *DRD1 *5' flanking region showed that multiple putative binding sites for transcription factors Sp1, AP1 and AP2 were detected, but no TATA and CAAT boxes were found in the presumptive promoter. By online prediction, 5 mutations in the 5' flanking region were found to be related with mutations of transcription factor binding sites. A-570G led to a GATA-1 binding site to disappear. A-647G induced the change from a SRY binding site to a HSF binding site. C-684A induced the change from a SRY site to HFH-3, Croc, Hb, CdxA binding sites. C indel located at -1157 generated a putative HSF binding site. G-1735C resulted in the loss of an ADR1 binding site and the gain of an E2F site.

### Genotype and Haplotype Structure

Seven SNPs in the coding region of the chicken *DRD1 *gene were genotyped in the NDH population. No polymorphism was detected in A+505G, whereas, in each of the other 6 SNPs, three genotypes were found in this population. Haplotype structure analysis showed that there were 2 haplotype blocks for the 6 SNPs. The corresponding base combinations for each haplotype block were shown in Table 5. Block 1 was composed of G+123A and T+198C and three haplotypes with frequencies higher than 1%, including H2 (AC, 21.28%), H3 (GT, 70.49%) and H4 (GC, 7.44%), were observed. Block 2 comprised G+1065A and C+1107T. Three haplotypes with frequencies higher than 1%, E2 (AC, 57.47%), E3 (GT, 18.26%), and E4 (GC, 24.11%), were found in this block.

**Table 5:** The corresponding base combinations for two haplotype blocks

Block 1 Haplotype	G+123A	T+198C	Frequency	Block 2 Haplotype	G+1065A	C+1107T	Frequency
H1(AT)	A	T	0.0079	E1(AT)	A	T	0.0016
H2(AC)	A	C	0.2128	E2(AC)	A	C	0.5747
H3(GT)	G	T	0.7049	E3(GT)	G	T	0.1826
H4(GC)	G	C	0.0744	E4(GC)	G	C	0.2411

### Association of Polymorphisms in the DRD1 Coding Region with Chicken Egg Production and Broodiness Traits

The G+123A was significantly associated with chicken broody frequency (P < 0.05). Furthermore, the EN values of chickens with the AA genotype were significantly higher than those with the GG genotype (P < 0.05) (Table 6). C+1107T was in significant association (P < 0.05) with chicken broody frequency (Table 7). No significant association was found in the other 4 markers (A-179T, T+198C, C+765T, and G+1065A) with chicken egg production and broodiness traits (P > 0.05).

**Table 6:** Association of the G+123A with egg production traits and broody traits in Ningdu Sanhuang Chickens

Traits^1^	P value	GG^2 ^(385)	AG^2 ^(217)	AA^2 ^(31)
AFE(d)	0.58	135.7 ± 0.6^a^	136.3 ± 0.7^a^	137.3 ± 1.9^a^
EN	0.10	113.3 ± 1.5^a^	114.6 ± 1.9^ab^	124.2 ± 4.9^b^
QEN	0.17	109.8 ± 1.5^a^	110.3 ± 1.8^a^	119.2 ± 4.8^a^
OEN	0.14	3.6 ± 0.3^a^	4.3 ± 0.4^a^	5.0 ± 1.0^a^
EW (g)	0.89	45.8 ± 0.2^a^	45.7 ± 0.3^a^	45.5 ± 0.6^a^
DB(d)	0.35	7.9 ± 0.8^a^	7.0 ± 1.0^a^	4.4 ± 2.5^a^
Number of nonbroody chickens	/	186	116	22
Number of broody chickens	/	199	101	9
Broody frequency (%)	/	51.69	46.54	29.03
χ^2^value	< 0.05	6.58*		

^1^AFE = age of first egg; EN = total egg number from 90 to 300 d of age; QEN = total number of qualified eggs from 90 to 300 d of age; OEN = total number of oafish eggs from 90 to 300 d of age; EW = weight of first egg; DB = duration days of broodiness.^2^Least-square means ± standard errors (SE); Number in brackets referred to the number of tested chickens of each genotype. ^a, b^means within a row with no common superscript are different significantly (P < 0.05). * indicated P < 0.05. χ^2 ^_0.05_(df = 2) = 5.99.

**Table 7:** Association of the C+1107T with egg production traits and broody traits in Ningdu Sanhuang Chickens

Traits^1^	P value	CC^2 ^(403)	TC^2 ^(201)	TT^2 ^(13)
AFE(d)	0.24	136.0 ± 0.6	135.7 ± 0.7	140.7 ± 2.9
EN	0.27	113.6 ± 1.5	115.1 ± 2.0	125.5 ± 7.6
QEN	0.36	109.8 ± 1.4	110.9 ± 1.9	120.3 ± 7.4
OEN	0.37	3.7 ± 0.3	4.2 ± 0.4	5.3 ± 1.5
EW (g)	0.88	45.8 ± 0.2	45.8 ± 0.3	45.3 ± 0.9
Duration of broodiness (d)	0.85	7.5 ± 0.7	7.3 ± 1.0	5.3 ± 3.9
Number of nonbroody chickens	/	195	108	11
Number of broody chickens	/	208	93	2
Broody frequency (%)	/	51.61	46.27	15.38
χ^2^value	< 0.05	7.58*		

^1^AFE = age of first egg; EN = total egg number from 90 to 300 d of age; QEN = total number of qualified eggs from 90 to 300 d of age; OEN = total number of oafish eggs from 90 to 300 d of age; EW = weight of first egg; DB = duration days of broodiness.^2^Least-square means ± standard errors (SE); Number in brackets referred to the number of tested chickens of each genotype. ^a, b^means within a row with no common superscript are different significantly (P < 0.05). * indicated P < 0.05. χ^2^_0.05_(df = 2) = 5.99.

### Association of the Haplotypes with Chicken Egg Production and Broodiness Traits

A total of 623 individuals with 6 diplotypes (30 of H2H2, 178 of H2H3, 31 of H2H4, 324 of H3H3, 57 of H3H4, 3 of H4H4) were used in association analysis in the block 1. Significant association (P = 0.03) of the haplotypes of G+123A and T+198C with EW was observed. H2H4 had much lower value of EW (mean = 44.1 g) and was highly significantly different (P < 0.01) from H2H3, significantly (P < 0.05) from H3H4. Nevertheless, H2H2 had much higher value of EN (mean = 124.0) and QEN (mean = 118.9) and was significantly different from H3H3 (P = 0.04) and H4H4 (P = 0.02), respectively.

A total of 614 individuals with 6 diplotypes (200 of E2E2, 134 of E2E3, 174 of E2E4, 11 of E3E3, 67 of E3E4, 28 of E4E4) were used in association analysis in the block 2. The haplotypes of G+1065A and C+1107T were significantly associated (χ^2 ^value _(df = 5) _= 11.08, 0.01 < P < 0.05) with broody frequency. Nevertheless, E3E3 had much higher value of AFE (mean = 142.2) than other diplotypes and was significantly different with E2E2, E2E3, and E3E4 (P < 0.05).

### Tissue-specific Expression of the DRD1 and the mRNA Comparison between Broodiness and Non-broodiness Chickens

**Figure 1:**
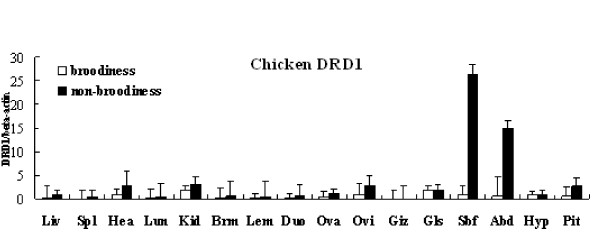
**The distribution of *DRD1 *mRNA in broodiness and non-broodiness chickens**. The horizontal axis and vertical axis indicate different tissues and 2^-ΔΔCt ^value (mean ± SEM), respectively. Liv = liver, Spl = spleen, Hea = heart; Lun = lung, Kid = kidney, Brm = breast muscle, Lem = leg muscle, Duo = duodenum, Ova = ovary, Ovi = oviduct, Giz = gizzard, Gls= glandular stomach, Sbf = subcutaneous fat, Abd = abdominal fat, Hyp = hypothalamus, Pit = pituitary.

The *DRD1 *mRNA was differentially expressed in distinct tissues (Figure 1). There was almost no mRNA present in gizzard. Low mRNA level was observed in liver, spleen, lung, breast muscle, leg muscle, as well as in duodenum. Instead, much higher levels of the *DRD1 *expression were detected in tissues such as heart, kidney, oviduct, glandular stomach, hypothalamus, and pituitary. Remarkably, the highest levels of the *DRD1 *expression were found in subcutaneous fat of non-broodiness chickens, and then abdominal fat.

In subcutaneous fat and abdominal fat, significantly difference of the *DRD1 *mRNA was found between broodiness and non-broodiness chickens (P < 0.05). The level of non-broodiness was 26 to 28 times higher than that of broodiness. The expression of non-broodiness was observed to be 5-fold greater than that of broodiness in pituitary. Also a prominent decrease from non-broodiness to broodiness was displayed in heart, oviduct, and kidney. In these tissues, the expression of non-broodiness was 2-3 times higher as compared to that of broodiness. The level of glandular stomach seen in non-broodiness was almost the same as in broodiness. Similarly, the same level was found in non-broodiness and broodiness hypothalamus (Figure 1).

## Discussion

In this study, abundant polymorphisms were found in the chicken *DRD1 *gene. Soller reported that SNP frequencies in poultry species ranged from 1:48 to 1:1632 bp [40]. Here SNP frequency of the chicken *DRD1 *gene was 1:115 bp and it was similar to previous study [41]. In this study, the absence of TATA and CAAT boxes was found in chicken *DRD1 *promoter, as reported in human [42]. Mutations in the promoter region can cause changes of transcription factor binding sites and consequently may affect the transcription and the phenotype [43]. In the 5' flanking region of the chicken *DRD1 *gene, there were multiple putative binding sites for transcription factor Sp1 and consensus sequences for AP1 and AP2 binding sites. It was consistent with the analysis of human *DRD1 *gene [44].

A variety of structural variations occurred in different regions of G protein coupled receptor proteins have been found to be related with diseases [45,46]. In the present study, one non-synonymous mutation (Ser169Gly) was present in the extracellular domain. As no polymorphism of this site was observed in the NDH population, its effects on chicken egg production and broodiness still required further study in other populations.

SNP in cytoplasmic tail and transmembrane I seemed to have great effects on egg production and broodiness. In this study, three mutations found within the cytoplasmic tail of the chicken *DRD1 *gene might cause the change of various functions even if they were synonymous. C+1107T, a mutation located in the cytoplasmic tail, was found to be associated with chicken broodiness, and haplotype analysis also provided similar results. In addition, G+123A, a variation in transmembrane I of the *DRD1 *gene, was associated with chicken egg production and broodiness traits. The polymorphisms of G+123A and C+1107T may be acted as Marker assistant selection (MAS) markers of reducing incidence of broodiness and improving egg production in modern poultry industry. In the same NDH population of our former study, two SNPs of the chicken *DRD2 *gene, A-16105G and T+619C, were also found to be significantly associated with broody frequency and duration of broodiness, respectively [47].

Other studies also indicated that the cytoplasmic tail of D1-like receptors, especially the N-terminal segment termed as the fourth intracellular loop, played a crucial role in the regulation of the activation of adenylyl cyclase, ligand binding, expression, and G protein coupling properties [48-52]. Members of G protein coupled receptors displayed considerable amino acid sequence conservation within transmembrane domains [53]. Through bioinformatics analysis, the presumed transmembrane domains of the *DRD1 *gene were proved to be highly conserved in diverse species. Many previous studies reported that some mutants present in transmembrane domains of the Dl receptor affect ligand interactions and receptor signal transduction [54-56]. It seemed that the variation may play a crucial role on egg production and broodiness traits by affecting ligand binding or signal transduction.

In mammals, the *DRD1 *gene was found to express in the tissues of striatum, nucleus accumbens, cerebral cortex, amygdale, olfactory tubercle, retina, limbic system, hypothalamus, and thalamus [57,58], but not in cerebellum, hippocampus, mesencephalon, pituitary, kidney, liver, lung and heart tissues [42,59,60]. In avian, it was revealed that the distribution of the *DRD1 *gene in the forebrain was substantially similar to that of mammals [61]. In this study, similar to mammals, high level of the chicken *DRD1 *mRNA was detected in the hypothalamus. However, high to moderate *DRD1 *mRNA were also detected in chicken heart, kidney, oviduct, glandular stomach, hypothalamus, pituitary, and adipose tissues and a considerably low but still detectable expression was found in liver, spleen, lung, muscle, and duodenum.

The level of the *DRD1 *mRNA was quantified in the brain of the domestic turkey hen during the reproductive cycle and it was expressed throughout the hypothalamus and pituitary [35]. But no significant difference of *DRD1 *mRNA abundance was observed in hypothalamic and pituitary throughout the reproductive cycle. Similarly, this study suggested that in hypothalamus, as well as in glandular stomach, the *DRD1 *mRNA levels seen in broody chickens were essentially the same as in non-broody ones. However, higher *DRD1 *mRNA content was found in pituitary (5-fold), heart (3-fold), oviduct (2.8-fold), and kidney (2-fold) of non-broody hens as compared to that of incubating hyperprolactinemic hens. In particular, it was interesting that there was a dramatic expression difference in adipose tissues from non-broodiness to broodiness stage. The level of the *DRD1 *mRNA in non-broody chickens was 26 to 28 times greater than that of broody chickens in adipose tissues including subcutaneous fat and abdominal fat. As chickens in broodiness had lower fatty content compared with non-broody chickens, the subtle decreased mRNA in broody chickens suggested that DRD1 was probably involved in fat deposition. In general, a high abundance of *DRD1 *mRNA was found in non-broodiness compared with broodiness in each tissue, except for the glandular stomach and hypothalamus. All these findings indicated that the *DRD1 *gene was probably related to chicken broodiness.

## Conclusions

In summary, the results of association analysis and the expression comparison of broody chickens with non-broody chickens demonstrated that the *DRD1 *had important effects on chicken egg production and broodiness incidence.

## Authors' contributions

HX carried out the mRNA research, analyzed the data and drafted the manuscript. XS contributed to the genotyping of most of the SNP, MZ and MF participated in the data analyses. HZ contributed to materials collection. QN and XZ contributed to the design of the study, the supervision of the study and the revision of this manuscript. All authors read and approved the final manuscript.
